# Supplementary material for: Plant Selection for the Establishment of Push–Pull Strategies for Zea mays–Spodoptera frugiperda Pathosystem in Morelos, Mexico
Source: Insects. 2020 Jun 4;11(6):349. doi: 10.3390/insects11060349 (PMC7349205; doi:10.3390/insects11060349)
Supplement: Supplementary file 1 [file insects-11-00349-s001.pdf]

insects

MDPI

Article

Plants selection for the establishment of push-pull strategies for *Zea mays - Spodoptera frugiperda* pathosystem in Morelos, Mexico

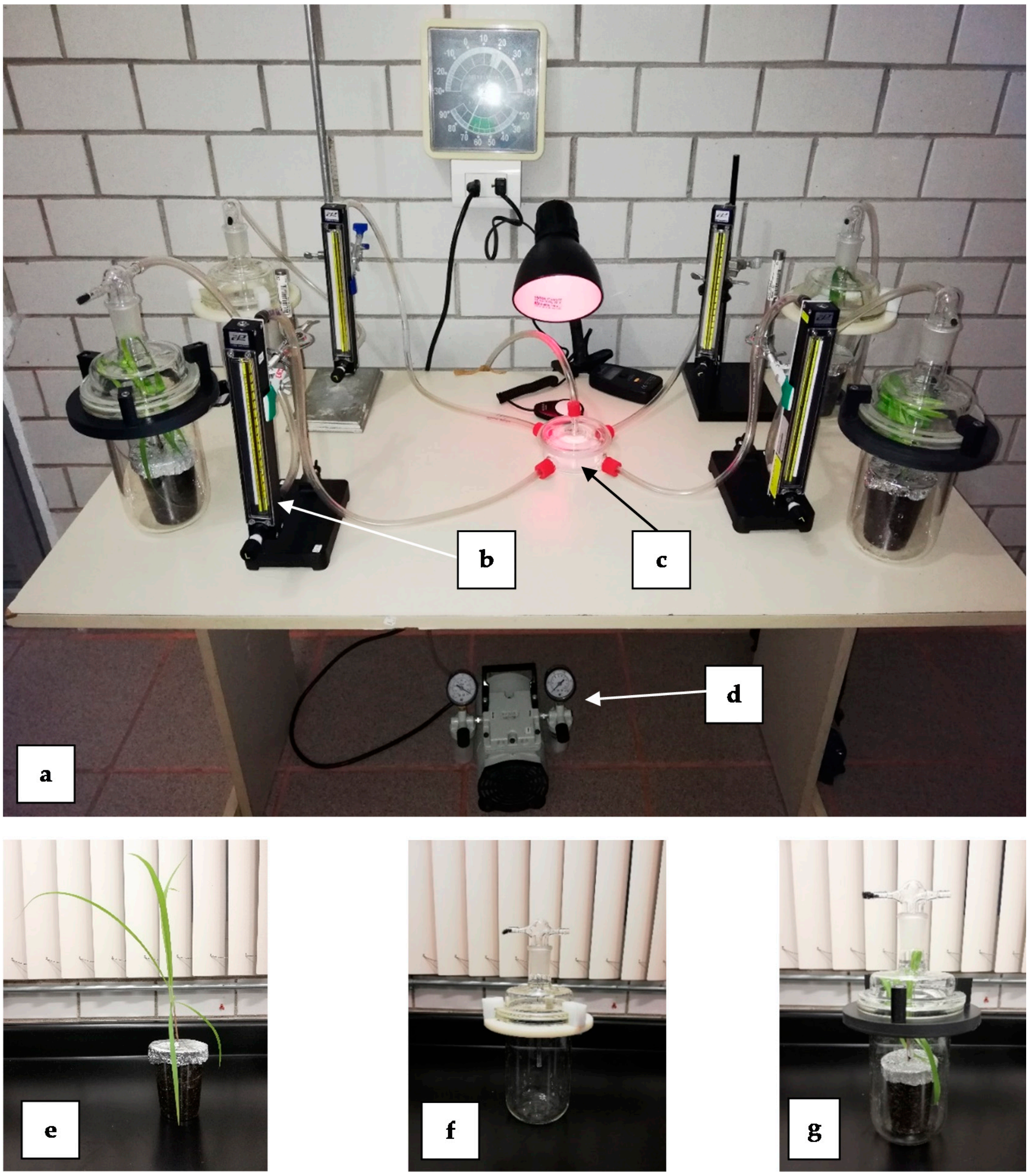

Figure S1. a. Four-way olfactometer set-up for testing olfactory response of L3 larvae of *S. frugiperda* to cues of potential attractant or repellent plants; **b.** Flowmeter; **c.** Central glass choice arena with four arms; **d.** Vacuum pump; **e.** Plant with aerial part isolated with aluminum foil; **f.** Air-tight glass chamber containing negative control (clean air); **g.** Air-tight glass chamber containing a plant (source of volatile compounds).

Table S1. Sigmoidal models adjusted for *S. frugiperda* larvae weight prediction in different grasses.

№	Authors	Mathematical expressions	Growth rate (dP/dt)	Inflection point
01	**Gompertz** [48]	$P_t = \beta_0 e^{-e^{\beta_1-\beta_2 t}} + \varepsilon$	$\beta_0\beta_2 e^{\beta_1-\beta_2 t} e^{-e^{\beta_1-\beta_2 t}}$	$t^* = \frac{\beta_1}{\beta_2}$ $P(t^*) = \frac{\beta_0}{e}$
02	**Richards** [49]	$P_t = \frac{\beta_0}{(1+e^{\beta_1-\beta_2 t})^{1/\beta_3}} + \varepsilon$	$\frac{\beta_0\beta_2 e^{\beta_1-\beta_2 t}}{\beta_3(1+e^{\beta_1-\beta_2 t})^{\frac{1}{\beta_3}+1}}$	$t^* = \frac{\beta_1}{\beta_2} - \frac{ln\beta_3}{\beta_2}$ $P(t^*) = \frac{\beta_0}{(1+\beta_3)^{1/\beta_3}}$
03	**Logistic** [50]	$P_t = \frac{\beta_0}{(1+\beta_1 e^{-\beta_2 t})} + \varepsilon$	$\frac{\beta_0\beta_1\beta_2 e^{-\beta_2 t}}{(1+\beta_1 e^{-\beta_2 t})^2}$	$t^* = \frac{ln\beta_1}{\beta_2}$ $P(t^*) = \frac{\beta_0}{2}$

P_t: Larval weight (g) at an age t (days); β_0: asymptotic value that the larvae weight can reach; β_1: parameter without biological importance; β_2: relative measure of larval weight growth rate; β_3: parameter that expresses the shape of the curve by the location of the inflection point; ε: random error.

Table S2. Mean (± SEM) trichome density of the abaxial surface of three sections of 30-day-old grass leaves in Yautepec, Morelos, Mexico.

Species	Number of trichomes/cm²		
	**Base**	**Medium**	**Apex**
B. hybrid cv. Mulato II	87.95 ±8.65 Aa	74.60 ±8.31 ABa	59.30 ±5.25 Ba
B. brizantha	53.95 ±4.09 Ab	21.45 ±1.86 Bb	28.65 ±2.91 Bb
P. maximum cv. Mombasa	1.45 ±0.20 Bc	5.00 ±0.53 Ac	1.65 ±0.36 Bc
P. maximum cv. Tanzania	0.80 ±0.19 Ac	0.15 ±0.08 Bc	0.05 ±0.05 Bc
S. sudanense	3.05 ±0.53 Ac	2.50 ±0.46 Ac	1.70 ±0.38 Ac
L. multiflorum	0.90 ±0.19 Ac	0.85 ±0.18 Ac	0.65 ±0.21 Ac
Z. mays	2.10 ±0.35 Ac	2.35 ±0.36 Ac	1.75 ±0.38 Ac

Means followed by the same letter (uppercase in rows and lowercase in columns) do not differ from each other for Tukey test (α=0.05).
